# Conditional performance factors in padel players: a mini review

**DOI:** 10.3389/fspor.2023.1284063

**Published:** 2023-11-09

**Authors:** J. Guijarro-Herencia, E. Mainer-Pardos, H. Gadea-Uribarri, A. Roso-Moliner, D. Lozano

**Affiliations:** ^1^ Health Sciences Faculty, University San Jorge, Zaragoza, Spain; ^2^ Faculty of Education, Pontifical University of Salamanca, Salamanca, Spain

**Keywords:** racket sports, internal load, external load, heart rate, stroke

## Abstract

**Introduction:**

Padel's global growth highlights its technical complexity. The first publications focus on the physical aspects of padel, using methods that evaluate players’ endurance, strength, speed, and range of motion, while the quantification of load, using internal and external indicators, is crucial for performance optimization, whilst taking into account athletes’ profiles and levels. Therefore, the objective entailed analyzing the scientific literature about the conditional demands in competition within padel players from different levels.

**Methods:**

Data was collected from various databases and after the selection process, the information was analyzed in mini review.

**Results:**

The mini review shows that the conditional demands are categorized into internal and external load to try to obtain reference values that may define the demands of padel based on the competitive level and sex. Regarding the internal load, the heart rate (HR)% of the padel players from different levels is around 70%–80% of the HR Max. Regarding the external load, in femalés categories, a greater number of strokes are made per point which entails a longer duration in the points.

**Conclusions:**

Padel research usually analyses physical demands using internal and external loading. HR% values (70%–80% HR Max) are consistent across studies. There is variation between variables such as strokes per point, and contextual factors affect the metrics. Further exploration is vital to obtain comprehensive benchmarks and understand the demands of this sport.

## 1. Introduction

Padel has experienced a remarkable worldwide growth in recent years, becoming a popular sport with more than 18 million players and more than 300,000 registered licenses. It is present in more than 90 countries, overseen by the International Padel Federation, which encompasses 51 national federations (1). The complexity of padel as a sport are centered on its technical intricacies, which require well-established tactical strategies, endurance for physically demanding matches and the ability to avoid unforced errors along with those of the teammate (2, 3).

Previously, there has been scarcity in research relevant to the physical performance in Padel. One of the first authors to introduce methods to assess a player's physical condition through various tests targeting factors that influence performance was Sanchez-Pay et al. (4). This involved assessments of endurance, strength, speed, and flexibility (4).

Quantification of loads represents a fundamental factor in optimizing athletes’ performance. This involves monitoring physiological and performance adaptation, both during training sessions and competitions (5). Load quantification uses two main indicators: internal load and external load (6). Internal load measures the specific requirements of each individual by assessing physiological variables such as heart rate (HR) and lactate levels (7). In contrast, external load measures the overall demands of training or competition by recording absolute values such as action time, strokes and distance covered. These indicators are influenced by the level, age, and sex of the athletes, which requires an approach based on individual profiles and different skill levels or categories.

Other racket sports have focused their results on other fields of interest: anthropometric profiling, physiology, and physical performance (heart rate, maximum oxygen consumption, and lactate), biomechanics, injury epidemiology and match analysis (8, 9).

For all these reasons, we see the need to conduct this research to review the latest scientific publications on the subject, with the aim of helping coaches and physical trainers to have reference data for their training.

Following the aforementioned reasons, the objective of this study was to analyze the existing scientific literature in relation to the physical demands imposed on padel players in different competitive categories.

## 2. Materials and methods

**Figure 1:**
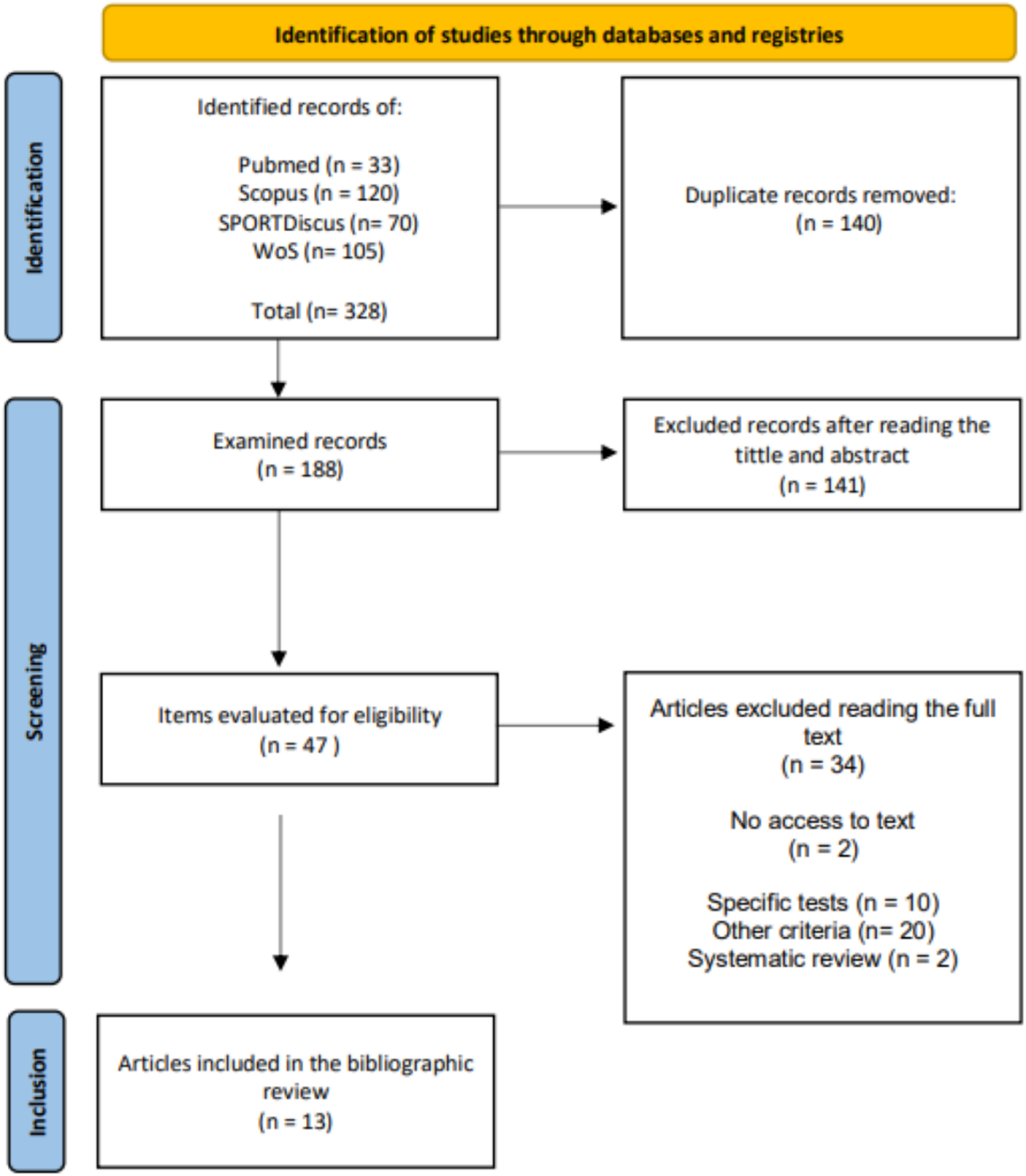
Flowchart.

This mini review was conducted following the PRISMA (Preferred Reporting Items for Systematic Reviews and Meta-Analyses) methodology, updated in 2020 to include the latest advances in methods for evaluating scientific studies (Figure 1). Eligibility for this review focused on studies directly related to conditional factors in paddle tennis, a relatively new sport with limited research, given its recent global emergence.

### 2.1. Search strategy

The guidelines for systematic review were used to search for articles meeting the inclusion criteria in four databases: Web of Science, PubMed, Scopus and SPORTDiscus.

### 2.2. Inclusion criteria and exclusion criteria

The search was conducted between January 2013 and until June 2023. The search strategy was: (Pádel[ti] OR Padel[ti] OR Paddle tennis[ti]). These words were searched in title, abstract and keywords. Full-text reports in English and Spanish were considered for the analysis. Studies were selected that met the following inclusion criteria: articles published between 2013 and June 2023, articles in English and Spanish, studies that analyzed male and female players of any age, of any level, studies conducted in real competition or in simulated situations, and studies that evaluated physiological variables such as heart rate, blood lactate concentration, rate of perceived exertion and game variables such as match duration, set duration, game duration, point duration and number of strokes.

The exclusion criteria were as follows: abstracts, conferences and/or congress communications, systematic reviews, meta-analyses, articles on pathologies or adapted sport.

Data from all investigations were collected according to the time variables selected by the researchers that were framed within the competition, based on the complete assessment of matches, sets, games or points. Only articles in which data were taken during competitions were included and those in which data were taken in laboratory tests or conditioned situations outside the match structure were excluded.

Regarding the analysis of the conditional factors, studies traditionally analyze the internal load, considering variables such as HR, categorized into average heart rate (HR Med), maximum heart rate (HR Max) and percentage of heart rate (HR %) and the subjective perception of exertion (RPE). On the other hand, for the analysis of the external load, variables such as duration and/or rest times were analyzed, as well as technical-tactical elements such as hits, always considering the temporal structure of the competition (point, game, set, match).

## 3. Results and discussion

Once all the studies had been compiled, with a total of 328, those articles that were duplicated were eliminated. The remaining articles were filtered by title and abstract to read in depth those that could add value to the study. All studies that did not evaluate conditional demands and were outside the context of competition were excluded. Those that met these requirements were reviewed one by one until a total of 13 definitive articles were collected.

A total of 231 padel players, men (*n* = 161) and women (*n* = 70) of all competition levels participated in the 13 selected studies (10–22). The competitive level is divided into professional (*n* = 120), national (*n* = 40), regional (*n* = 23) and minors (*n* = 32). The competitive level of the athletes is classified according to the indications of McKay et al. (23).

### 3.1. Internal load

The analysis and synthesis of this review shows six studies with data on the internal load of padel players in different categories (Table 1). All of them register HR Med, with the amateur level being the lowest data recorded for this variable (126.8 ± 10.4 bpm) (20) compared to the data presented by male athletes in the 1st national category (159.1 ± 13.8 bpm) (14). On the other hand, it has been found that the FC Med values vary greatly depending on the study for athletes in the same category. Another study with male subjects for 1st national of 131.7 ± 16.3 bpm while for the 2nd national category they show 156.4 ± 15.6 bpm and finally values of 150.8 ± 14.4 bpm for 3rd national (17) which is far from other records with similar categories of the papers collected for the study. Even the 151 ± 8.1 bpm and 153.7 ± 14.6 bpm, registered in the professional level for groups of male and female subjects (10, 14) closely resemble some data obtained by lower level. This may be due to the fact that in padel there are moments in which one of the players does not hit the ball, has less relative activity time and that is why compared to other sports such as badminton, HR Med is much lower (24). Our results show four articles that collect HR Max in which the group with the lowest level of regional 3rd category presents values of 154.8 ± 7.3 bpm (20), values similar to the values of the highest-level male subjects, who show the highest records with 186.6 ± 15.2 bpm in professional male subjects and 188.6 ± 5.9 bpm in 1st national male subjects (14). HR Max is very similar to other racket sports such as tennis or badminton (4, 24–26). The similarity in HR Med data between male and female players at the professional level and some lower levels could indicate that certain physiological thresholds are maintained at different skill levels. This could also be due to the inherent nature of paddle tennis, in which not all players are always actively participating. However, it is important to underline that, compared to other racket sports such as badminton, the FC Med of padel seems significantly reduced. This could be an area of intrigue for both coaches and athletes, who wonder whether it is necessary to increase the intensity in training scenarios or whether it is the nature of padel that demands such results.

**Table 1:** Internal load in padel.

Author	Study	*N*	Sex	Age (years)	Level	HR Med (beat/min)	HR Max (beat/min)	HR % (beat/min)	RPE	LA (mmol)
Castillo-Benitez et al. (17)	Transverse	8	M	28.7 ± 6.76	1ª National	131.7 ± 16.3	–	69.0 ± 6.9	3.2 ± 2.0	2.6 ± 1.3
		8	M		2ª National	156.4 ± 15.6	–	81.3 ± 7.8	5.9 ± 1.7	2.7 ± 1.4
		8	M		3ª National	150.8 ± 14.4	–	78.0 ± 7.1	5.1 ± 1.7	3.4 ± 1.8
Pradas et al. (10)	Transverse	6	F	28.2 ± 0.6	Professional	151 ± 8.1	177 ± 9.3	76.3	–	2.4 ± 0.7
Carbonell et al. (18)	Transverse	9	F	32.8 ± 12.3	1ª/2ª/3ª Nat.	150 ± 8.6	179 ± 9.4	78.6 ± 3.6	–	–
Diaz et al. (20)	Transverse	8	M	22.5 ± 1.1	3ª Regional	126.8 ± 10.4	154.8 ± 7.3	65.7	–	–
Llin-Mas et al. (14)	Transverse	7	M	31.1 ± 5.9	Professional	153.7 ± 14.6	186.6 ± 15.2	85.9	–	–
		7	M	25.4 ± 3.8	1ª National	159.1 ± 13.8	188.6 ± 5.9	86.4	–	–

HR Med, medium heart rate; HR Max, maximum heart rate registered on court; HR %, heart rate percentage of maximum heart rate; LA, blood lactate concentration; RPE, rating of perceived exertion; Beat/Min, beats per minute.

The RPE is only discussed by one article (17) and it shows that the subjects with the highest level are those with the lowest subjective perception effort with 3.2 ± 2.0 compared to 5.1 ± 1.7 for those in the lower level. This could be attributed to the fact that more experienced players possess a deeper understanding of the sport, employing superior tactical awareness to successfully navigate specific situations. In contrast, lower level players tend to make more unforced errors, resulting in shorter intervals between goals. Players who demonstrate good technical skills, but fail to achieve success, often have to amend their technical and tactical errors, which is more physically demanding. The RPE values observed in tennis are higher with figures of 7.9 ± 1 in official matches and 7.1 ± 1.2 in simulated matches (26, 27), while in badminton it shows values of 15.7 ± 1.7 on a scale of 6–20 (28). The inclusion of the RPE alone in one item and the notable difference between high and low level players indicate that experience, knowledge of tactics and game strategies could play a key role in how players identify their exertion levels. This suggests that coaches and players may need to focus on the tactical and technical aspects of the game to better manage their energy expenditure.

As for lactate values only two studies acknowledge them. The highest values of LA concentration in blood are equivalent to 3rd national level subjects with 3.4 ± 1.8 mmol by 2.7 ± 1.4 mmol in 2nd national level subjects and 2.6 ± 1.3 mmol, in 1st national level subjects all of them male (17). The other, studies female professional paddle athletes obtaining values of 2.4 ± 0.7 mmol (10). One of the main reasons may be the physical preparation to which they are subjected since all professional players who participated in the study trained a minimum of nine hours per week plus physical work. Interestingly, the fact that male 3rd national level subjects present higher lactate concentrations than female professional paddle tennis players or even male 1st national level subjects could indicate variations in anaerobic contributions during play. This discrepancy could be due to different training regimes or physical preparation, suggesting the need for physical conditioning programs adapted to the different competitive categories.

### 3.2. External load

The analysis and synthesis show eight studies collected for this analysis consider data in relation to the external load in padel players of different categories, ages, levels and sex (Table 2). The duration in seconds of the structures of the sport of padel divided into points, games, set and match is suited in eight of the nine articles.

**Table 2:** External load in padel.

				Age (years)		MatchD (s)		SetD (s)		GameD (s)		PointD (s)		Strokes		
Author	Study	*N*	Sex		Level	Total	Real	Total	Real	Total	Real	Total/Real	TBP	Match	Set	Point
Sánchez-Alcaraz et al. (16)	Cross-sectional	16	M	14.24 ± 1.86	Minors	–	–	1,745.2 ± 646.7	532.2 ± 345.2	–	–	9.23 ± 8.14	14.12 ± 9.24	–	–	6.7 ± 7.5
Torres-Luque et al. (11)	Cross-sectional	8	M	18 ± 35	Pro Top 10	3,041.8 ± 263.3	1,050.2 ± 170	–	–	–	–	9.3 ± 4	10.2 ± 9.3	–	–	9.3 ± 1.1
		8	F			3,721.3 ± 774.8	1,453.1 ± 260.5	–	–	–	–	9.7 ± 4.8	12.3 ± 9.5	–	–	9.5 ± 2.2
Garcia-Benitez et al. (12)	Cross-sectional	18	M	32.6 ± 5.1	Pro	5,029.2 ± 1,848.6	1,441.8 ± 521.4	2,111.4 ± 606	633 ± 183.6	159.6 ± 104.4	66 ± 34.8	10.8 ± 7.7	17.2 ± 7.7	1,178.9 ± 443.1	–	7.7 ± 6.3
		10	F	31.3 ± 4.1		5,344.6 ± 1,569.6	1,950 ± 690	2,296.8 ± 708.6	867 ± 316.8	216.6 ± 126.6	103.8 ± 66	15.8 ± 12.7	20.3 ± 7.2	1,338.8 ± 480.7	–	9.7 ± 8.3
Muñoz et al. (19)	Cross-sectional	15	M	25.4 ± 4.2	Regional	–	1,484 ± 174.6	–	684.9 ± 118.2	–	69.6 ± 35.7	12.7 ± 10.1	15.0 ± 6.3	–	–	–
Courel-Ibañez et al. (13)	Cross-sectional	9	F	32.8 ± 12.3	1st/2nd/3rd National	–	–	–	–	–	–	9.4 ± 7.2	–	–	–	–
García-Benitez et al. (22)	Cross-sectional	8	M	15.5 ± 1.1	U16	4,866 ± 954	1,740 ± 54	2,017 ± 455	1,866 ± 144	142 ± 89	61.6 ± 6.9	8.9 ± 6.1	14.3 ± 7.9	995 ± 194	–	6.1 ± 5.0
		8	F			4,926 ± 1,380	1,944 ± 216	2,035 ± 599	2,004 ± 210	167 ± 86	44.4 ± 8.7	11.3 ± 9.2	15.6 ± 6.1	986 ± 349	–	6.9 ± 6.4
		8	M		U18	5,214 ± 2,145	2,082 ± 84	2,166 ± 600	2,130 ± 126	163 ± 84	46.9 ± 7.3	12.0 ± 8.7	15.5 ± 6.4	1,185 ± 760	–	8.0 ± 6.2
		8	F			3,168 ± 1,002	2,088 ± 258	1,535 ± 650	1,824 ± 414	168 ± 107	41.7 ± 12.6	11.7 ± 9.1	14.1 ± 5.2	713 ± 281	–	7.2 ± 6.1
Lupo et al. (15)	Cross-sectional	14	M	–	Pro	–	–	–	–	–	–	12.6 ± 2.1	–	–	–	9.6 ± 1.5
		9	F	–		–	–	–	–	–	–	16.8 ± 2.8	–	–	–	12.2 ± 2.0
Sanchez-Alcaraz et al. (21)	Cross-sectional	12	M	34.1 ± 5.4	Professional	5,661 ± 1,665	1,855.8 ± 840	2,460 ± 360	–	–	–	12.6 ± 10	–	–	–	–
		12	F	30.3 ± 5.6		5,368.8 ± 1,620	1,680 ± 480	2,684.4 ± 821.4	–	–	–	13.5 ± 10	–	–	–	–

MatchD, match duration; SetD, set duration; GameD, game duration; PointD, point duration.

It can be seen that there are no relevant records for the total duration of a point, with the U16 male category presenting the lowest values with 8.9 ± 6.1 s (22) together with the other subjects in the minor categories with values of 9.2 ± 8.1 s (16) compared to values of professional male padel that register durations of 9.3 ± 4 s (11), 10.8 ± 7.7 s (12), 9.4 ± 7.2 s (13), 12.6 ± 2.1 s (15) and 12.6 ± 10 s (21) compared to the values of professional female padel that record 9.7 ± 4.8 s (11), 13.5 ± 10 s (21) and 16.8 ± 2.8 s (15). The duration of the points in women's padel is somewhat longer than that of the masculine and the reason could be because in men's padel there is more definition of power shots such as the smash. The weather conditions and the type of court can be a key factor as well in the measurement of data of this type of game. The duration of a point in padel is very similar to that recorded in tennis (25, 29) while in badminton the points are somewhat shorter (24). The time between points varies depending on each study and the values range from 10.2 ± 9.3 s registered in the professional male padel (11) to 20.3 ± 7.2 s registered in professional female padel (12). Each match is unique due to varying conditions and players with their specific on-court habits. Most articles document durations consistent with a standard padel match, keeping in mind that the rules allow up to twenty seconds between points. The variation in rest time between points across studies and categories could reflect individual player habits or playing strategies. Coaches could take these differences into account when planning players’ rest and recovery during matches.

On the contrary, only three articles refer to the total and real time of the duration of a game. The total duration of a game in professional male padel is 159.6 ± 104.4 s of which 66 ± 34.6 s are real game time for the total 216.6 ± 126.6 s of the female professional padel compared to the real 103.8 ± 66 s (12). Regarding the duration of a game in the male minors stage, it is 142 ± 89 s total compared to the 61.6 ± 6.9 s of real duration in the U16 category and 163 ± 84  s total compared to the 46.9 ± 7.3 real seconds for the U18 category. For the female U16 category it is 167 ± 86 s in total for 44.4 ± 8.7 real seconds and for the U18 category it is 168 ± 107 s total compared to 41.7 ± 12.6 real seconds (22).

Regarding the duration of a set, six articles collect data, four of which provide records of the total and real duration. If we talk about the total duration of a set of professional male padel, the table shows values of 2,111.4 ± 606 s and 2,460 ± 360 s respectively (12, 21) while the duration of a set for the professional female padel shows 2,296.8 ± 708.6 s and 2,684.4 ± 821.4 s (12, 21). In minor stages, the records are somewhat lower with 1,745.2 ± 646.7 s of total time and 532 ± 345.2 s for males with a mean age of 14.2 ± 1.9 years (16). For male padel players, the total duration of a set is 2,017 ± 455 s for the actual 1,866 ± 144 s in the U16 category, while for the U18 category the total values are 2,166 ± 600 s for the actual 2,130 ± 126 s. The total duration of a set for the female U16 category is 2,035 ± 599 s for the 2,004 ± 210 s of real time and values for the U18 female category of 1,535 ± 650 s of total time for the 1,824 ± 414 s of real time (12). In the female professional padel, the total and real duration of a set is somewhat greater compared to the male one. However, for the minor stage there is a substantial difference with a specific group such as the female U18 compared to the other values obtained. As it is such a small group (*n* = 8) the results obtained may not be representative or may be matches that are rarely played or of a very different level. The duration of matches and sets presents disparate results. Although professional women's padel matches appear to last longer than their male counterparts, the inconsistency of the data, especially at the lower levels, calls for further study. The varied total and actual match lengths could be influenced by numerous external factors, underscoring the importance for coaches to recognize these differences in order to adapt training sessions more effectively. Finally, it would be necessary to collect more data and be able to compare them to obtain more enlightening data.

Of the nine articles that allude to the external load, four of them take total and real values of the duration of a complete game. Three articles use this indicator with respect to professional padel and the values recorded for the male category are 3,041.8 ± 263.3 s of total time for the real 1,050.2 ± 170 s (11),5,029.2 ± 1,848.6 s total for the 1,441.8 ± 521.4 s of real time (12) and 5,661 ± 1,665 s total compared to the 1,855 ± 840 s (21) while for the female professional padel the values obtained are 3,721.3 ± 774.8 s of total match time and 1,453.1 ± 260.5 s of real time (11), 5,344±,6 ± 1,569,6 s total for the 1,950 ± 690 s of real time (12) and values of 5,368,6 ± 1,620 s of total time that become 1,680 ± 480 s of real time (21). The other article that assesses the total and real duration of a complete padel match does so in the minor stage and the total seconds of the U16 and the U18 category are 4,866 ± 954 and 5,214 ± 2,154 s total for the 1,740 ± 54 and 2,082 ± 84 s of real time respectively. The data obtained from the total duration of a match in the U16 and U18 female categories are 4,296 ± 1,380 and 3,168 ± 1,002 s total compared to the real 1,944 ± 216 and 2,088 ± 258 s (22). There is a great disparity in terms of the total and real values obtained in the duration of a match and it may be due to different factors such as the weather, the track, the type of players, etc. However, what is not collected in the study and it is not valued is the number of sets played and that can be a key differentiating factor since the time of a match can vary enormously depending on whether a match is closed in two or three sets.

The number of hits is another indicator of external load in the sport of padel and there are five articles that collect information regarding the total number of hits throughout a point, set and match. In the professional category, it is recorded that the male category makes an average of 9.3 ± 1.1 hits, 7.7 ± 6.3 and 9.6 ± 1.5 hits per point for the 9.5 ± 2.2 hits, 9.7 ± 8.3 and 12.2 ± 2 hits made in the female category (11, 12, 15). It can be seen that the male average moves around eight or nine hits per point in professional padel while in the female category there is greater variability in terms of the number of hits per point. However, it would be good to be able to compare with other articles where more data is collected. The other two articles collect the number of hits per point in minor stages, with 6.7 ± 7.5 being the total number of hits recorded by the subjects who only belonged to the male category (16) while the second article for the U16 and U18 male category records 6.1 ± 5 and 8 ± 6.2 hits compared to 6.9 ± 6.4 and 7.2 ± 6.1 hits in the female category (12). The average number of strokes in tennis varies between 2.7 and 5.3 strokes per point, depending on the type of court as well as the level and sex, according to (26, 29, 30). Only two articles collect data on total hits throughout a padel match, in which the professional level male category adds a total of 1,178.9 ± 443.1 hits for the 1,338.8 ± 480.7 of the female professional category (12) while the second article does it in the minor stage where 995 ± 194 and 1,185 ± 760 hits are recorded in the U16 and U18 male category for the 986 ± 349 and 713 ± 281 hits recorded for the female category respectively (22). How many spikes occur per set is a point of curiosity, since the number of sets played in a match could mean a significant change in the number of total spikes recorded. On the other hand, there are hardly any comparable records of these parameters for the external load, so no clear conclusion can be drawn since the categories, ages and results are not comparable. Furthermore, comparison of these data with tennis provides a unique perspective on the relative physical demands of each sport, which helps coaches to develop sport-specific conditioning programs. However, a larger set of comparable data across categories and ages is crucial to draw more definitive conclusions and inform training practices more thoroughly.

The results highlight the differences in internal load and external load between sex and training levels in paddle tennis. These findings are crucial to adapt and optimize the training and physical preparation programs of male and female padel players according to their sex and competitive category. Furthermore, they highlight the importance of considering the specificity of this intermittent sport when designing training strategies and sports planning.

The present study has some limitations. As it is such a young sport and is not that popular in many places yet, there are hardly any articles that offer comparable data. In recent years padel has evolved a lot, so even with already existing articles, the values and obtained results become obsolete with the most modern padel in which the physical factor is increasingly relevant, which makes it a much faster sport than a few years ago. Regarding the articles that exist, each one measures what it considers or prioritizes for each parameter and variable, so it is difficult to compare one with another. As the years go by, more articles will appear, and it will be easier to find verified information. Padel is a sport with a lot of variability, not only by category and sex, but also has many different conditions. An important limitation is the lack of consideration of atmospheric conditions as they could influence certain physiological parameters of the players such as heart rate or blood lactate accumulation. There are many different strokes and many uncertain situations which are difficult to record in a particular categorical way. Finally, many of the variables cannot be compared with each other except with data obtained within the same group with a similar level of players. To address these limitations, future systematic reviews could employ a more structured approach to study selection, ensuring better alignment in parameters and variables. Future research on paddle tennis should take into account the potential impact of weather conditions on player performance. In addition, as the popularity of the sport increases, collaboration among researchers could facilitate the creation of a standardized set of metrics for the study of paddle tennis, thus contributing to its comparability.

## 4. Conclusions

The most used grouping by the scientific literature specialized in padel to analyze the physical demands during competitions are internal load and external load. Considering the HR% as a measure of the internal load, there are no great differences in the values collected in the different studies. The HR% of the padel players of different categories is around 70%–80% of the HR Max. Regarding the external load generally in female categories, a greater number of hits per point are made. Some of the variables measure different approaches, so the data obtained cannot be compared, while in others, such as the distance covered, there is hardly any data that can serve as a reference for this parameter in terms of the external load in padel. It is essential to recognize the multifaceted nature of padel competitions, characterized by a multitude of situational factors that generate fluctuations in the metrics recorded. Further exploration of these contextual elements is warranted to establish comprehensive benchmarks for each category and skill level. Finally, investigation of these contextual elements will aid in the understanding of the physical demands of the sport and help coaches and physical trainers to have reference data for their training and competition.
